# Influence of Dietary Supplementation With a Powder Containing A.N. ProDen™ (*Ascophyllum Nodosum*) Algae on Dog Saliva Metabolome

**DOI:** 10.3389/fvets.2021.681951

**Published:** 2021-06-22

**Authors:** Jerzy Pawel Gawor, Jacek Wilczak, Ulla K. Svensson, Michal Jank

**Affiliations:** ^1^Klinika Arka, Krakow, Poland; ^2^Institute of Veterinary Medicine, Department of Physiological Sciences, Warsaw University of Life Sciences, Warsaw, Poland; ^3^UKS Life Science Consulting AB, Lund, Sweden; ^4^Institute of Veterinary Medicine, Department of Pre-Clinical Sciences, Warsaw University of Life Sciences, Warsaw, Poland

**Keywords:** ascophyllum, dogs, metabolomics, metabolites, oral health, saliva

## Abstract

The objective of this placebo-controlled, double-blind, randomized study (designed according to evidence-based medicine standards) was to determine the effect of 30-day administration of powdered brown algae, *Ascophyllum nodosum* (ProDen PlaqueOff, SwedenCare AB, Sweden), on saliva metabolomes in dogs. Sixty client-owned dogs underwent professional dental cleaning and were randomly subdivided into two groups receiving daily powdered brown algae *A. nodosum*, or a placebo (microcrystalline cellulose in powder), adjusted to their bodyweight. After a comprehensive oral health assessment and professional dental cleaning, which were both performed under general anesthesia, clinical assessments for gingivitis, plaque, and calculus were conducted. Saliva samples were collected at Day 0 and Day 30 of supplementation. Whole saliva is a mixed fluid that is derived predominantly from the major salivary glands but it also contains numerous other constituents. Additionally, its composition varies on whether salivary secretion is basal or stimulated. Authors put efforts to avoid contamination of saliva by other constituents and character of saliva was basal. Quadrupole time-of-flight (QTOF) mass spectrometer was used to conduct analysis of the saliva samples. Metabolomic analyses identified clear changes after 30 days of supplementation, and the direction of these changes was completely different than in dogs that received a placebo treatment during the same period. The positive clinical effect of 30 days of *A. nodosum* supplementation on oral health status in dogs described in previous publication combined with the absence of some metabolites in the saliva of dogs on day 30 of supplementation suggest that brown algae inhibit or turn off some pathways that could enhance plaque or calculus development. The exact mechanism of *A. nodosum* is still unclear and warrants further study.

## Introduction

Periodontal disease is one of the most common conditions in dogs. It has a prevalence ranging from 44 to 100% in adult dogs, and its incidence and severity increase with age (1). It is initiated by plaque formation and calculus deposition on the tooth enamel, which leads to gingivitis, periodontitis, and tooth loss (2). The majority of cases are irreversible but often controllable (1). There are many different approaches to periodontal disease prevention, the majority of which involve oral care and the elimination of plaque and calculus.

One approach to periodontal disease prevention in dogs is the use of powdered *Ascophyllum nodosum* seaweed as a food additive (3). These brown algae can be administered to dogs directly as a food additive or indirectly as an ingredient of pet food, treats, or other nutritional products. In a previous study, treatment of adult dogs with edible treats containing *A. nodosum* for 90 days reduced plaque deposition by 40% and calculus formation by 20% compared to a control group (3). A formula containing the active agent as a powder has also been evaluated in dogs and received the Veterinary Oral Health Council Seal of Acceptance (www.vohc.org). Additional studies are currently being conducted in cats. In humans ingesting the algae significantly reduced the formation of supragingival calculus and plaque, and the occurrence of gingival bleeding (4).

The composition of saliva and identification of saliva metabolites which play a role in oral health and disease are the subject of intense research. In humans, due to deficiency of periodontal screening procedures the use of metabolomic tools has led to identification of metabolites associated with periodontal variables linked with tissue destruction, host defense mechanisms and bacterial metabolism (5). Quantitatively, production of saliva or flow rates are either considered basal or surge. Salivary composition varies, depending on whether salivary secretion is basal or stimulated (6). Whole saliva is a mixed fluid that is derived predominantly from the major salivary glands. However, whole saliva also contains constituents that do not originate in the salivary gland, including gingival crevicular fluid (GCF), serum transudate from the mucosa and sites of inflammation, expectorated bronchial and nasal secretions, microorganisms and products, viruses, fungi, desquamated epithelial cells, and food and debris. Depending on their size and charge, molecules from blood enter into saliva (7).

Despite evidenced effectiveness of brown algae administration for the reduction of plaque and calculus deposition, the exact mechanisms of its action are currently unknown. One suggested mechanism is based on our hypothesis that some of the seaweed's active ingredients are absorbed in the small intestine and then secreted into the oral cavity via saliva. That is why to investigate our hypothesis, in the study presented here, the metabolomes of non-stimulated, whole saliva were investigated in dogs that received powdered *A. nodosum* in their food for 30 days.

## Materials and Methods

### Dogs

The dogs included in the study were client-owned. All procedures performed during the study were conducted in accordance with standard veterinary care protocols and relevant Polish regulations (Art.1 ust.2 pkt1 Dz. U.2015 poz. 266), so the study did not require ethics committee approval. All dog owners provided written informed consent for the participation of their dogs in the study. The study adhered to VOHC requirements to assess the efficacy of an oral supplement containing *A. nodosum*.

### Inclusion/Exclusion Criteria

None of the dogs included in the study had received any anti-inflammatory drugs, or antibiotics within the 30 days prior to the initiation of the treatment. All dogs were required to have most of their teeth, but the teeth specifically required for the assessment were bilateral maxillary I3, C, P3, P4, and M1 and mandibular C, P3, P4, and M1. Oral health status was required to be no worse than PD2 as determined via American Veterinary Dental College stage (www.avdc.org). The parameters assessed to determine the general health status of the dogs included urinalysis, complete blood count (CBC), thyroxine, selected serum tests (urea, total protein, albumin, glucose, alanine transaminase, alkaline phosphatase), and a clinical assessment (see section Dental procedures and oral health assessment for further details). Pregnant dogs, and any dogs with non-gingival inflammation oral ulceration or laceration as well as with systemic disease or increased levels of thyroxine were excluded. The latter was due to increased amount of iodine in supplement (the seaweed) and this information is included in manufacturer's description.

The randomization lists for each trial and group were generated using the Research Randomizer (8) randomization program. The numbers of dogs receiving the active substance or a placebo were equal (*n* = 30 per group). The clients were blinded to group assignment, as were the personnel conducting the clinical study and the statistician. None of the dogs received any form of oral hygiene treatment during the study other than the study preparation or the corresponding placebo.

### Products

During the study, all dogs received the supplement in powder form prepared by the manufacturer (Swedencare AB, Sweden). One group received powder containing the brown algae A.N. ProDen™ (ProDen PlaqueOff^®^), and the other group received a microcrystalline cellulose powder. One measuring scoop included 330 mg of product, and the recommended daily doses were 0.5 of a scoop for dogs weighing 2.0–4.9 kg, 1.0 scoop for dogs weighing 5.0–9.9 kg, and 1.5 scoops for dogs weighing 10.0–14.9 kg. This results in a dose of product in the range of 33.2–82.5 mg/kg. The two products (active and placebo) were packed into a neutral non-transparent container that included a measuring scoop. The containers were labeled with the study details and the date, but not with an overt indication of whether the container contained the experimental preparation or the placebo. During the study period all dogs were fed dry kibble (Hill's SP Canine Adult Chicken) and tap water was provided *ad libitum*. Information about food intake and any variations were recorded by the dog owners.

### Dental Procedures and Oral Health Assessment

On the first day of the study (T0) all participating dogs were treated at the dental facility of Arka Veterinary Clinic in Krakow, Poland, which is a referral clinic with a hospital, training facility, and dental/surgery profile (www.arka-vet.pl). The study procedures conducted at T30 were performed under sedation at the same facility. The study was conducted between May 2018 and August 2018.

Animals qualified for anesthesia on T0 if they had an anesthesia risk of ≤ 1 (9). All dogs were first evaluated via a general physical examination that included visual assessment, heart and lung auscultation, capillary refill time assessment, and body weight and body temperature measurements. All parameters and remarks were recorded on an anesthesia chart. Intravenous catheters were placed and blood was collected for immediate laboratory work. Urine samples were collected via cystocentesis and immediately evaluated using a UA analyzer to measure pH and specific gravity, and test for protein, glucose, ketones, and blood. The Oral Health Index (OHI) of each dog was evaluated before sedation in accordance with published protocols, and was defined as the sum of scores obtained for three parameters; lymphadenopathy, dental deposits, and periodontal disease, with 0 points indicating optimal oral health, and 6 points indicating the poorest oral health (10).

Sedation was performed using recommended doses of medetomidine (Sedator, Eurovet Animal Health B.V.) and butorphanol (Torbugesic, Zoetis, Poland Sp. z o.o.). Preoxygenation was then performed using a mask for the delivery of medical oxygen. After sedation was achieved, each dog was administered the recommended dose of propofol (Scanofol, ScanVet, Poland Sp. z o.o.) to induce general anesthesia. An endotracheal tube was placed, the cuff was filled, cardio monitor peripheral probes were attached to the dog's body, and a temperature maintenance system was installed. General anesthesia was maintained via isoflurane and oxygen for the duration of dental procedures, which included comprehensive oral health assessment with periodontal probing, dental charting, and full mouth radiography.

The oral assessment methods for gingivitis, plaque, and calculus were applied to 9 (5 + 4) target teeth on each side; maxillary 13, C, P3, P4, and M1, and mandibular C, P3, P4, and M1. Every dog had 18 teeth assessed. The buccal surfaces of the target teeth on both sides of the mouth were scored by an experienced assessor who evaluated gingival bleeding index, plaque, and calculus in that order.

### Saliva Sampling

All dogs were fasted and were not engaged in any activities for at least 12 h prior to awake examination, during which a saliva sample was collected. According to the VOHC requirements, scoring episode was performed within +/−3 h of when the next treatment would have been given if the trial had continued. The dogs were given the Ascophyllum product in the afternoon, the rechecks and thus the saliva collection was approximately 3 h prior to normal feeding time so the sampling was 21 h prior to the next food or supplement administration.

A sterile size 80 paper point ISO was introduced into the oral vestibulum space via dental forceps and kept there until it was completely soaked with saliva. The average time of soaking was 15 s and approximately amount of saliva that made complete soaking was 25 μl. At a time of collection samples, the completion of soaking was based on time and visual assessment. It then was placed into a labeled sterile Eppendorf tube and frozen at −20°C. If required, this process was also performed on the contralateral side. The tubes were then packaged with ice and transported to the laboratory. Saliva samples were taken from all dogs at two timepoints; prior to professional dental cleaning (T0), and at least 12 h after the last of 30 days of administration of the experimental product or placebo (T30). Saliva collection was performed before sedation and prior to the assessment of dental indices.

### Metabolomic Analyses

#### Equipment

Metabolomic analyses were performed using a high-pressure liquid chromatography Symbiosis Pico UhPLC system. The detector used was a SCIEX TripleTOF 5600+ DuoSpray Source for SCIEX TripleTOF 5600+ (TurboIonSpray and APCI). Data were analyzed using SCIEX MarkerView™, XCMSplus, and MetaboAnalyst 4.0 software.

#### Sample Preparation

Filters with samples were placed into 1.5-mL tubes, and then, 800 μL of a 1:1 mixture of acetonitrile and methanol was added. Vials were vortexed (2,000 rotations for 15 min) and centrifuged for 15 min at 13,000 rpm. Supernatant was transferred to glass autosampler vials, which were then placed in an autosampler at 4°C. Saliva samples (8.0 μL) were injected directly into a Spark Holland Symbiosis™ Pico. Chromatographic separation was performed on Hypersil chromatographic column, BDS C18, 150 × 4.6 mm, 5 mm with a Hypersil C18 guard column (10 × 2.1 mm, size 5 μm). The mobile phase consists of methanol: formic acid (99:1, v/v) A and water: formic acid (99:1, v/v) B, the flow rate was constant 500 μLmin−1. Gradient elution of mobile phase 100% A was started, 1.1–40 min linear gradient to 100% B, 40.1–55 min 100% B, 55.1–60 min linear gradient to 100% A. The runtime of the method was 60 min.

MS Parameters: The optimized detection conditions included curtain gas (N2) 25 psi, nebulizer gas (N2) 20 psi, heater gas (N2) 50 psi, ion source voltage floating 5,500 V, and source temperature 500°C. Samples with a heated electrospray ionization probe measured in positive ionization (H-ESI+). Every third analyzed saliva sample using the Calibrant Delivery System (SCIEX) MS sytem was auto-calibrated using original calibrators (SCIEX).

#### Calculations and Metabolites Identification

Metabolomic comparisons were performed within each experimental group between T0 and T30. Metabolite profiles obtained in the 100–1100 Da range with 5 cps sensitivity were analyzed using MarkerView™ software to compare the groups, including Student's *t*-tests and principal component analysis (PCA). The generated metabolomics profiling data sets were processed by the control software of the Analyst^®^ mass spectrometer and saved in a specific data format (*raw). The first step was to convert data from Excalibur-specific raw files to open format files (*mzXML) using MS Convertor software (MSConvert). Subsequently, metabolomics data were processed using the XCMSplus platform. Additionally, principal component analysis (PCA) resulted in comparative profiles of metabolomes of specific groups were analyzed by MarkerView™ software. The classification of identified metabolites in particular metabolic pathways was conducted using the XCMSplus platform and MetaboAnalyst 4.0 software with a probability threshold of *p* < 0.0001. The identification of metabolites was carried out on the basis of the ChemSpider database (access via PeakView™) indications above 80% probability of confirming a given structure - these data were compared with the indications of metabolites from the MetaboAnalyst 4.0 database.

## Results

**Figure 1:**
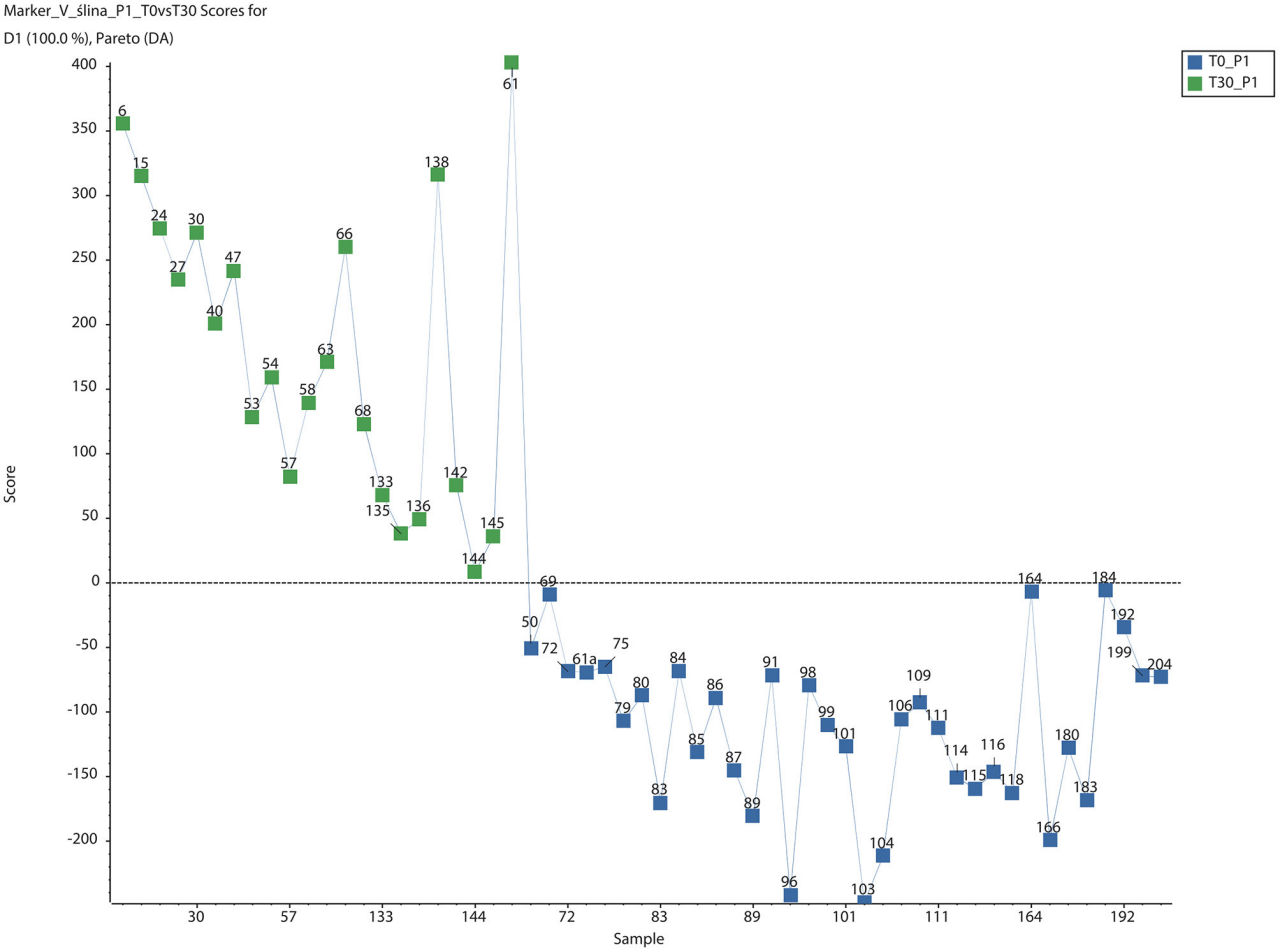
PCA-DA Score plots for saliva of dogs after 30-day-long period of placebo administration. Green boxes and blue boxes represent metabolome of sample from dogs in day 0 and day 30 after placebo administration, respectively (PCA-DA, Scaling PARETO, SCIEX MarkerView™).

**Figure 2:**
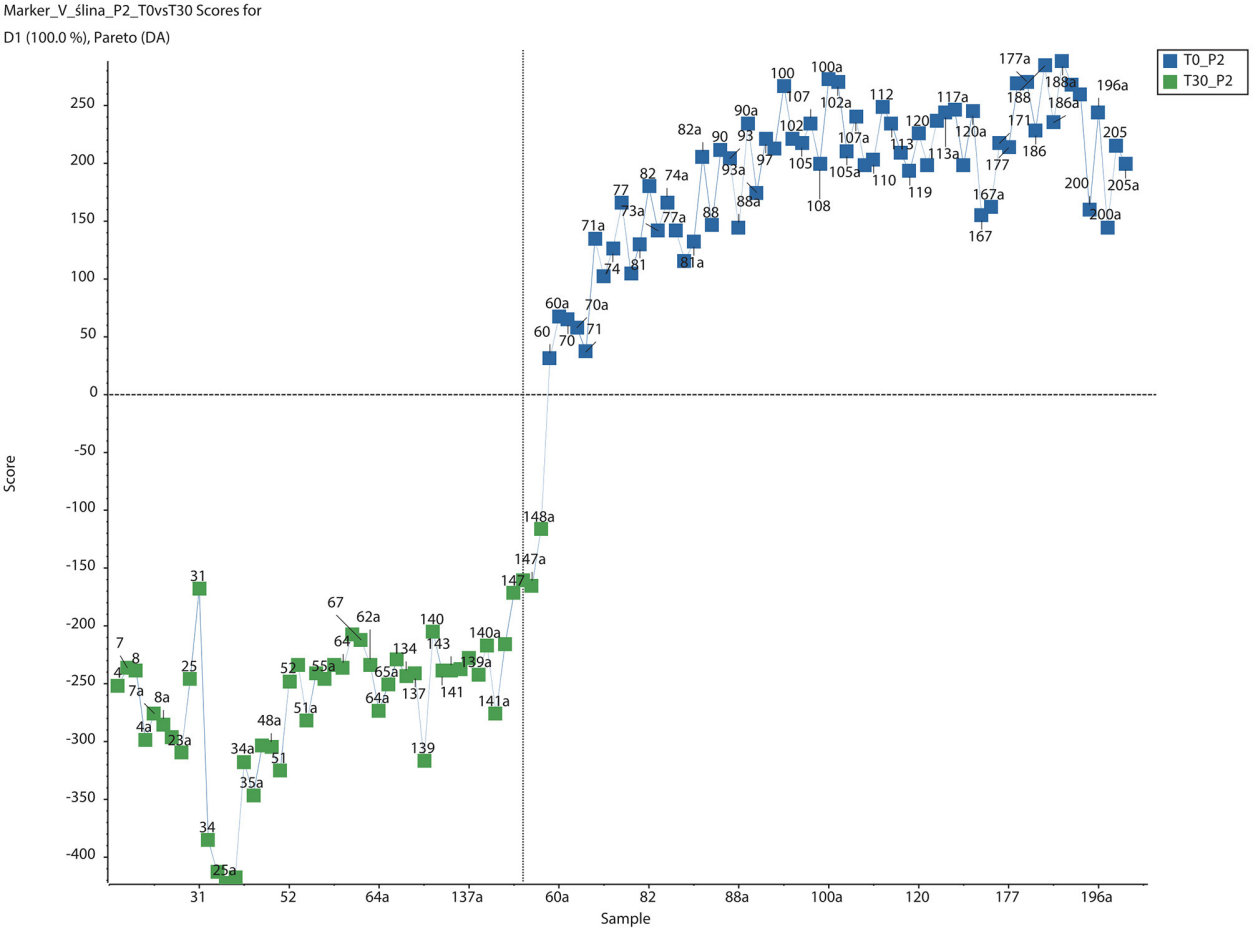
PCA-DA Score plots for saliva of dogs after 30-day-long period of *Ascophyllum nodosum* administration. Green boxes and blue boxes represent metabolome of sample from dogs in day 0 and day 30 after *Ascophyllum nodosum* administration, respectively (PCA-DA, Scaling PARETO, SCIEX MarkerView™).

After 30 days of product administration there were significant changes in dog saliva metabolomes (Figures 1, 2). In the untreated control group 4,347 metabolites exhibited levels that differed on day 30 compared to day 0. Of these, 962 differed significantly (*p* > 0.05) (data not shown). Using specialized software, 68 different molecules were identified based on their molecular weights. Based on a list of known metabolites included in software database, no significantly altered metabolic pathways were identified after 30 days of placebo treatment (Table 1). Analyses performed on individual metabolites revealed three groups; metabolites elevated after 30 days of administration, metabolites decreased after 30 days of administration, and metabolites absent after 30 days of administration (Table 2). The absent metabolites included molecules involved in metabolism of vitamin A, as well as metabolites of some drugs (tamoxifen). Metabolites that had increased after 30 days of placebo administration included molecules involved in arachidonic acid metabolism, leukotriene metabolism, and prostaglandin formation from arachidonate.

**Table 1:** List of metabolic pathways identified in dog saliva after 30 days of placebo administration.

**Pathway**	**Total**	**Hits (all)**	**Hits (sig.)**	***p*-value**	**Gamma P**
Drug metabolism - cytochrome P450	53	20	3	0.489	0.120
*De novo* fatty acid biosynthesis	106	21	3	0.524	0.125
Purine metabolism	80	13	2	0.516	0.139
Glycerophospholipid metabolism	156	24	3	0.619	0.139
Tyrosine metabolism	160	20	2	0.754	0.181
Vitamin A (retinol) metabolism	67	33	3	0.823	0.183
C21-steroid hormone biosynthesis and metabolism	112	60	5	0.910	0.203
Leukotriene metabolism	92	41	3	0.919	0.223
Prostaglandin formation from arachidonate	78	50	2	0.993	0.347
Arachidonic acid metabolism	95	65	2	0.999	0.420

**Table 2:** List of significantly (*p* < 0.05) regulated metabolites in dog saliva after 30 days of placebo administration.

**No**.	**Metabolite**	**Metabolic pathway**	**KEGG ID**	**Molecular weight**	***p*-value**	**Fold Change (FC)**	**logFC**	**Remarks**
1	(4Z,7Z,10Z,13Z,16Z,19Z)-docosahexaenoic acid	*De novo* fatty acid biosynthesis	C06429	328.4,880	0.014	57.703	1.761	
2	N(omega)-(ADP-D-ribosyl)-L-arginine	Vitamin B3 (nicotinate and nicotinamide) metabolism	C01201	715.5,014	<0.001	3.714	0.570	
3	1-(1-alkenyl)-sn-glycero-3-phosphate	Glycerophospholipid metabolism	C15646	197.1,030	0.001	3.603	0.557	
4	S-succinyldihydrolipoamide	TCA cycle	C01169	307.4,294	0.031	298.063	2.474	
5	7-methyluric acid	Caffeine metabolism	C16355	182.1,368	<0.001	2.777	0.444	
6	1-methyluric acid	Caffeine metabolism	C16359	474.6,511	<0.001	2.777	0.444	
7	(9E)-octadecenoic acid	Fatty acid activation	C01712	282.2,559	0.001	2.037	0.309	
8	(9Z)-octadecenoic acid	*De novo* fatty acid biosynthesis	C00712	282.4,614	0.001	2.037	0.309	
9	Hypoxanthine	Purine metabolism	C00262	136.1,115	0.034	2.028	0.307	
10	(5Z,8Z,11Z,14Z,17Z)-eicosapentaenoic acid	*De novo* fatty acid biosynthesis	C06428	302.4,510	<0.001	1.594	0.202	
11	Homovanillic acid	Tyrosine metabolism	C05582	182.1,733	0.005	1.543	0.188	
12	16-glucuronide-estriol	Androgen and estrogen biosynthesis and metabolism	C05504	464.5,055	0.005	1.543	0.188	
13	L-fucose 1-phosphate	Fructose and mannose metabolism	C02985	244.1,364	0.316	1.541	0.188	
14	Pregnanolone	C21-steroid hormone biosynthesis and metabolism	C05480	318.4,935	0.316	1.541	0.188	
15	1,2-naphthoquinone	Xenobiotics metabolism	C14783	158.1,534	0.006	1.457	0.163	
16	11-cis-retinal	Vitamin A (retinol) metabolism	C02110	284.4,357	0.006	1.457	0.163	
17	N6,N6,N6-trimethyl-L-lysine	Lysine metabolism	C03793	188.2,673	0.006	1.457	0.163	
18	Leukotriene E4	Leukotriene metabolism	C05952	439.6,086	0.006	1.457	0.163	
19	1-(5′-phosphoribosyl)-5-amino-4-(N-succinocarboxamide)-imidazole	Purine metabolism	C04823	454.2,833	0.006	1.457	0.163	
20	Hydroxytamoxifen	Drug metabolism - cytochrome P450	C05011	387.5,140	0.006	1.457	0.163	
21	Phenylacetic acid	Tyrosine metabolism	C07086	136.1,479	0.004	1.445	0.160	
22	Sterol	Glycerophospholipid metabolism	C00370	248.4,036	0.193	0.838	−0.077	Negative value of logFC = lower metabolite content at T30
23	(2R,3S)-2,3,4-trihydroxybutanoic acid	Ascorbate (vitamin C) and aldarate metabolism	C01620	136.1,033	0.001	0.480	−0.318	Negative value of logFC = lower metabolite content at T30
24	Sterol	C21-steroid hormone biosynthesis and metabolism	C00370	284.4,036	0.001	0.408	−0.389	Negative value of logFC = lower metabolite content at T30
25	Lidocaine	Drug metabolism - cytochrome P450	C07073	234.3,373	<0.001	0.120	−0.922	Negative value of logFC = lower metabolite content at T30
26	Dechloroethylcyclo-phosphamide	Drug metabolism - cytochrome P450	C16550	198.5,877	<0.001	0.058	−1.237	Negative value of logFC = lower metabolite content at T30
27	6-mercaptopurine	Drug metabolism - other enzymes	C02380	152.1,771	<0.001	0.020	−1.695	Negative value of logFC = lower metabolite content at T30
28	Retinyl ester	Vitamin A (retinol) metabolism	C02075	313.4,538	<0.001			Metabolite is absent at T30
29	11beta-hydroxyprogesterone	C21-steroid hormone biosynthesis and metabolism	C05498	330.4,611	0.014			Metabolite is absent at T30
30	16(R)-HETE	Arachidonic acid metabolism	C14778	320.4,663	0.006	1.457	0.163	
	11,12-EET	Arachidonic acid metabolism	C14770	320.4,663	0.004	1.445	0.160	
	14,15-EET	Arachidonic acid metabolism	C14771	320.4,663	0.004	1.445	0.160	
	19(S)-HETE	Arachidonic acid metabolism	C14749	320.4,663	0.004	1.445	0.160	
	20-HETE	Arachidonic acid metabolism	C14748	320.4,663	0.004	1.445	0.160	
	20-hydroxy arachidonic acid	Glycerophospholipid metabolism	C14748	320.4,663	0.004	1.445	0.160	
	5,6-EET	Arachidonic acid metabolism	C14768	320.4,663	0.004	1.445	0.160	
	8,9-EET	Arachidonic acid metabolism	C14769	320.4,663	0.004	1.445	0.160	
31	17alpha-hydroxyprogesterone	C21-steroid hormone biosynthesis and metabolism	C01176	330.4,611	0.316	1.541	0.188	
	11-deoxycorticosterone;	C21-steroid hormone biosynthesis and metabolism	C03205	330.4,611	<0.001	0.120	−0.922	Negative value of logFC = lower metabolite content at T30
32	5(S)-HETE	Arachidonic acid metabolism	C04805	336.4,657	<0.001	10.476	1.020	
	5(S)-HPETE	Leukotriene metabolism	C05356	336.4,657	0.006	1.457	0.163	
	Leukotriene B4	Leukotriene metabolism	C02165	336.4,657	0.006	1.457	0.163	
	Prostaglandin A1	Prostaglandin formation from arachidonate	C04685	336.4,657	0.006	1.457	0.163	
	Prostaglandin B1	Prostaglandin formation from arachidonate	C00959	336.4,657	0.006	1.457	0.163	
	15(S)-HETE	Arachidonic acid metabolism	C05966	336.4,657	0.004	1.445	0.160	
	Prostaglandin C1	Prostaglandin formation from arachidonate	C04686	336.4,657	0.004	1.445	0.160	
	15H-11,12-EETA;	Arachidonic acid metabolism	C14781	336.4,657	<0.001	0.020	−1.695	Negative value of logFC = lower metabolite content at T30

*The identification of metabolites was carried out on the basis of the ChemSpider database (access via PeakView™) indications above 80% probability of confirming a given structure so the table contains also the names of isomers or molecules with the same molecular weight with the same p-values and FC*.

In the group treated with the *A. nodosum* preparation 4,213 metabolites exhibited levels that differed on day 30 compared to day 0. Of these, 1,570 metabolites differed significantly (*p* < 0.05) (data not shown). Using specialized software 61 different molecules were identified based on their molecular weights. Based on a list of known metabolites, no significantly altered metabolic pathways were identified after 30 days of *A. nodosum* treatment (Table 3), although the significance of changes within the selenoamino acid metabolism pathway was considerably high (*p* = 0.063). The number of metabolites absent in dog saliva after 30 days of *A. nodosum* administration was higher than that in the control group (Table 4). The administration of *A. nodosum* was associated with a complete absence of 27/61 identified metabolites in the dog saliva. These absent metabolites included molecules involved in androgen and estrogen biosynthesis and metabolism (dehydroepiandrosterone), and prostaglandin synthesis from arachidonate and fatty acid metabolism (linoleic acid). Metabolites increased in dog saliva after *A. nodosum* administration included molecules involved in selenoamino acid metabolism (selenomethionine Se-oxide; tetrahydrofolate), linoleate metabolism, and squalene and cholesterol biosynthesis.

**Table 3:** List of metabolic pathways identified in dog saliva after 30 days of *Ascophyllum nodosum* administration.

**No**.	**Pathway Name**	**Total**	**Hits (all)**	**Hits (sig.)**	***p*-value**	**Gamma P**
1	Selenoamino acid metabolism	35	6	3	0.067	0.0,489
2	Tyrosine metabolism	160	28	6	0.350	0.068
3	Fatty acid metabolism	63	19	4	0.421	0.081
4	Drug metabolism - other enzymes	31	6	2	0.277	0.084
5	Vitamin E metabolism	54	21	4	0.504	0.091
6	Fatty acid activation	74	35	6	0.580	0.094
7	Porphyrin metabolism	43	15	3	0.494	0.097
8	Methionine and cysteine metabolism	94	9	2	0.478	0.110
9	Purine metabolism	80	17	3	0.584	0.110
10	Squalene and cholesterol biosynthesis	55	27	4	0.714	0.125
11	Pyrimidine metabolism	70	11	2	0.592	0.128
12	Urea cycle/amino group metabolism	85	12	2	0.641	0.136
13	Aspartate and asparagine metabolism	114	12	2	0.641	0.136
14	Vitamin A (retinol) metabolism	67	29	4	0.767	0.138
15	Linoleate metabolism	46	23	3	0.789	0.152
16	Drug metabolism - cytochrome P450	53	15	2	0.762	0.163
17	Xenobiotics metabolism	110	15	2	0.762	0.163
18	*De novo* fatty acid biosynthesis	106	34	4	0.867	0.170
19	Glycosphingolipid metabolism	67	17	2	0.822	0.181
20	Carnitine shuttle	72	21	2	0.902	0.215
21	Bile acid biosynthesis	82	23	2	0.929	0.232
22	Androgen and estrogen biosynthesis and metabolism	95	45	4	0.968	0.243
23	Glycerophospholipid metabolism	156	29	2	0.973	0.281
24	D4 & E4-neuroprostane formation	37	32	2	0.984	0.305
25	Leukotriene metabolism	92	44	3	0.989	0.305
26	C21-steroid hormone biosynthesis and metabolism	112	60	4	0.996	0.347
27	Prostaglandin formation from arachidonate	78	50	3	0.996	0.347

**Table 4:** List of significantly (*p* < 0.05) regulated metabolites in dog saliva after 30 days of *Ascophyllum nodosum* administration.

**No**.	**Metabolite**	**Metabolic pathway**	**KEGG ID**	**Molecular weight**	***p* value**	**Fold Change (FC)**	**logFC**	**Remarks**
1	L-palmitoylcarnitine	Carnitine shuttle	C02990	399.6,077	<0.001	9.073	0.958	
2	7-alpha-Hydroxy-3-oxo-4-cholestenoate	Bile acid biosynthesis	C17337	430.6,200	<0.001	817.951	2.912	
3	2-(S-glutathionyl)acetyl glutathione	Xenobiotics metabolism	C14863	654.6,678	0.005	4.411	0.655	
4	alpha-tocopherol	Vitamin E metabolism; Ascorbate (Vitamin C) and aldarate metabolism	C02477	430.7,061	0.004	4.402	0.644	
5	N6,N6,N6-trimethyl-L-lysine	Lysine metabolism	C03793	188.2,673	0.004	39.033	1.591	
6	2′-deoxyadenosine 5′-diphosphate	Purine metabolism; Pyrimidine metabolism; Hexose metabolism	C00206	411.2,017	0.030	2.529	0.403	
7	Docosahexaenoic acid	*De novo* fatty acid biosynthesis	C06429	328.4,883	<0.001	2.122	0.327	
8	N1-acetylspermine	Aspartate and asparagine metabolism	C16681	244.3,769	<0.001	18.137	1.259	
9	N-carbamoyl-L-aspartate	Pyrimidine metabolism	C00438	176.1,274	<0.001	16.801	1.225	
10	N(omega)-(ADP-D-ribosyl)-L-arginine	Vitamin B3 (nicotinate and nicotinamide) metabolism	C01201	715.5,014	<0.001	11.332	1.054	
11	Glycochenodeoxycholate	Bile acid biosynthesis	C05466	449.6,233	<0.001	1.896	0.278	
12	Selenomethionine Se-oxide	Selenoamino acid metabolism	C05708	212.1,057	0.001	1.873	0.273	
13	Geranylgeranyl diphosphate	Squalene and cholesterol biosynthesis	C00353	450.4,432	0.008	1.636	0.214	
14	20-hydroxyleukotriene E4	Leukotriene metabolism	C03577	455.6,080	0.177	1.293	0.112	
15	Lithocholic acid	Bile acid biosynthesis	C03990	376.5,726	0.030	1.289	0.110	
16	3′-phosphoadenylylselenate;	Selenoamino acid metabolism	C05696	554.1,593	0.036	1.168	0.067	
17	2′-deoxyinosine 5′-phosphate	Purine metabolism	C06196	332.2,066	0.188	0.629	−0.201	Negative value of logFC = lower metabolite content at T30
18	Codeine-6-glucuronide	Drug metabolism - cytochrome P450	C16577	475.4,884	0.002	0.344	−0.463	Negative value of logFC = lower metabolite content at T30
19	SN38 glucuronide	Drug metabolism - other enzymes	C11376	568.5,287	0.003	0.030	−1.527	Negative value of logFC = lower metabolite content at T30
20	Protoporphyrinogen IX	Porphyrin metabolism	C01079	568.7,058	0.003	0.030	−1.527	Negative value of logFC = lower metabolite content at T30
21	5-acetylamino-6-formylamino-3-methyluracil	Caffeine metabolism	C16365	226.1,894	0.001			Metabolite is absent at T30
22	(9Z,11E,15Z)-(13S)-hydroperoxyoctadeca-9,11,15-trienoate	Omega-3 fatty acid metabolism	C04785	310.4,284	<0.001			Metabolite is absent at T30
23	Levomethadyl acetate	Drug metabolism - cytochrome P450	C08012	353.4,977	<0.001			Metabolite is absent at T30
24	7-dehydrodesmosterol	Squalene and cholesterol biosynthesis	C05107	382.6,218	<0.001			Metabolite is absent at T30
25	(R)-4′-phosphopantothenoyl-L-cysteine	Vitamin B5 - CoA biosynthesis from pantothenate	C04352	402.3,578	0.004			Metabolite is absent at T30
26	Squalene	Squalene and cholesterol biosynthesis	C00751	410.7,180	0.001			Metabolite is absent at T30
27	Diacetylchitobiose (chitobiose)	Sialic acid metabolism	C01674	424.4,003	<0.001			Metabolite is absent at T30
28	6-mercaptopurine	Drug metabolism - other enzymes	C02380	152.1,771	<0.001			Metabolite is absent at T30
	Thiopurine	Urea cycle/amino group metabolism	C01756	152.1,771	<0.001			Metabolite is absent at T30
29	Phenylacetylglutamine	Tyrosine metabolism	C04148	264.2,771	0.076	1.688	0.227	
	Formyl-N-acetyl-5-methoxykynurenamine	Tryptophan metabolism	C05642	264.2,771	0.076	1.688	0.227	
30	Linoleic acid	Glycerophospholipid metabolism; Di-unsaturated fatty acid beta-oxidation	C01595	280.4,455	<0.001			Metabolite is absent at T30
	9-cis,12-cis-octadecadienoic acid	Fatty acid metabolism; Linoleate metabolism	C01595	280.4,455	<0.001			Metabolite is absent at T30
	Linoleic acid;	Fatty acid activation; *de novo* fatty acid biosynthesis	C01595	280.4,455	<0.001			Metabolite is absent at T30
31	9-cis-retinal	Vitamin A (retinol) metabolism	C02110	284.4,357	<0.001			Metabolite is absent at T30
	retinal-11-cis	Vitamin A (retinol) metabolism	C16681	284.4,357	<0.001			Metabolite is absent at T30
32	Dehydroepiandrosterone	C21-steroid hormone biosynthesis and metabolism	C01227	288.4,244	<0.001			Metabolite is absent at T30
	17-beta-hydroxy-4-androsten-3-one	Prostaglandin formation from arachidonate	C00535	288.4,244	<0.001			Metabolite is absent at T30
	5beta-androstane-3,17-dione	Prostaglandin formation from arachidonate	C03772	288.4,244	<0.001			Metabolite is absent at T30
	Dehydroepiandrosterone	Androgen and estrogen biosynthesis and metabolism	C01227	288.4,244	<0.001			Metabolite is absent at T30
	17-beta-hydroxy-4-androsten-3-one	Androgen and estrogen biosynthesis and metabolism	C00535	288.4,244	<0.001			Metabolite is absent at T30
	5-beta-androstane-3,17-dione	Androgen and estrogen biosynthesis and metabolism	C03772	288.4,244	<0.001			Metabolite is absent at T30
	5-alpha-androstane-3,17-dione	Androgen and estrogen biosynthesis and metabolism	C00674	288.4,244	<0.001			Metabolite is absent at T30
33	(9R,10S)-(12Z)-9,10-epoxyoctadecenoic acid	Linoleate metabolism	C14825	296.4,449	<0.001	49.832	1.698	
	(12R,13S)-(9Z)-12,13-epoxyoctadecenoic acid	Linoleate metabolism	C14826	296.4,449	<0.001	49.832	1.698	
	(9Z,11E)-(13S)-13-hydroxyoctadeca-9,11-dienoic acid	Linoleate metabolism	C14762	296.4,449	<0.001	49.832	1.698	
34	5-pregnen-3beta-ol-20-one	Androgen and estrogen biosynthesis and metabolism	C01953	316.4,776	<0.001			Metabolite is absent at T30
	5-beta-pregnane-3,20-dione	Prostaglandin formation from arachidonate	C00054	316.4,776	<0.001			Metabolite is absent at T30
	5-pregnen-3beta-ol-20-one	Prostaglandin formation from arachidonate	C01953	316.4,776				Metabolite is absent at T30
	20-alpha-hydroxy-4-pregnen-3-one	Prostaglandin formation from arachidonate	C04042	316.4,776	<0.001			Metabolite is absent at T30
35	Sphinganine 1-phosphate	Glycerophospholipid metabolism	C01120	381.4,877	<0.001			Metabolite is absent at T30
	Sphinganine 1-phosphate	Glycosphingolipid metabolism	C01120	381.4,877	<0.001			Metabolite is absent at T30
36	14-demethyllanosterol	Squalene and cholesterol biosynthesis	C05108	412.6,908	0.041	2.065	0.315	
	Isofucosterol	Squalene and cholesterol biosynthesis	C08821	412.6,908	0.041	2.065	0.315	
	7-dehydroavenasterol	Squalene and cholesterol biosynthesis	C15782	412.6,908	0.041	2.065	0.315	
37	Adenosine 3′,5′-bisphosphate	Tyrosine metabolism; Methionine and cysteine metabolism; Xenobiotics metabolism; Glycosphingolipid metabolism; Prostaglandin formation from arachidonate; Glycosphingolipid biosynthesis - ganglioseries; Keratan sulfate biosynthesis; Proteoglycan biosynthesis	C00054	427.2,011	0.012	1.852	0.268	
	2′-deoxyguanosine 5′-diphosphate	Purine metabolism; Pyrimidine metabolism;	C00361	427.2,011	0.012	1.852	0.268	
38	Tetrahydrofolic acid	Selenoamino acid metabolism	C00101	445.4,292	0.004	2.827	0.451	
	Tetrahydrofolate	Methionine and cysteine metabolism; Urea cycle/amino group metabolism; Histidine metabolism; Glycine, serine, alanine and threonine metabolism; Vitamin B9 (folate) metabolism	C00101	445.4,292	0.004	2.827	0.451	
39	Uroporphyrin I	Porphyrin metabolism	C05767	830.7,469	<0.001	0.109	−0.962	Negative value of logFC = lower metabolite content at T30
	Uroporphyrin III	Porphyrin metabolism	C02469	830.7,469	<0.001	49.832	1.698	

*The identification of metabolites was carried out on the basis of the ChemSpider database (access via PeakView™) indications above 80% probability of confirming a given structure so the table contains also the names of isomers or molecules with the same molecular weight with the same p-values and FC*.

## Discussion

The sum of the small molecules present in a biological sample, known as the “metabolome” has become an area of interest to many researchers in recent years. A consequence of this is the development of metabolomics, which can be described as untargeted measurement of the metabolome. The results of metabolomic analyses are influenced by genetics, environmental factors, and different clinical conditions that affect animals. This technology can be applied to human and animal clinical diagnostics, as well as nutrition, exercise, physiology, agriculture/plant biochemistry, and toxicology. One of the key factors supporting the metabolomics development is the possibility of identifying new potential biomarkers that could become standard diagnostic components in human and animal health screenings (11).

A metabolomic diagnostic approach has previously been applied in many different clinical contexts (12), and saliva as one of numerous body fluids is an obvious area of interest. In humans the composition of the saliva metabolome is a promising diagnostic tool for systemic diseases as well as oral problems, and investigation of the saliva metabolome has even resulted in the development of a specific field known as salivaomics (13). Studies in this area have yielded important information about caries, oral cancer, and systemic conditions (14–16). Due to the ease of sample collection the metabolomic study of saliva may become an important diagnostic tool in the future (17).

To the best of the authors' knowledge, the current study is one of the first to evaluate canine saliva with respect to metabolomics, particularly with regard to oral health. The reason for undertaking the study was to investigate the influence of supplementation of dogs' diets with *A. nodosum* algae on oral health status, which has been described in our previous studies (3). Clinical effects of supplementation were evident, but the mechanisms of *A. nodosum*'s action are still unclear. Because the available laboratory modalities (complete blood count, serum studies) did not reveal any significant changes in dogs receiving *A. nodosum*, we assumed that it may influence the composition of dog saliva.

To identify changes in dog saliva composition it was necessary to evaluate the whole saliva metabolome. Protocol for collection, storage, and transportation was designed based on a protocol used for nuclear magnetic resonance-based oral biofluid samples (18). The method used in the current study differed from that recently described by (19), but it facilitated the acquisition of informative samples. Comparative studies are necessary however, to identify the best method of saliva sampling from dogs for metabolomic analyses.

The analyses conducted in the present study yielded some important conclusions. First, in placebo group there were changes in saliva metabolome composition of saliva (Figure 1). Analysis of a list of differentially regulated metabolites revealed increasing levels of proinflammatory leukotrienes, and many different metabolites of arachidonic acid or prostaglandins formed from arachidonate (Table 1). Conversely, many metabolites involved in the metabolism of vitamin A had disappeared from dog saliva after 30 days of treatment. The exact meaning of these changes is unclear however the authors presume that it was oral prophylactic procedure that influenced the composition of saliva.

Secondly in the current study changes in the saliva metabolome were clearly evident in dogs after 30 days of *A. nodosum* administration (Figure 2), but the directions of these changes differed. An ingredient derived from algae, known as isofucosterol, was detected after 30 days of *A. nodosum* supplementation. It is one of the sterols produced solely by algae so its presence almost certainly resulted from the algae supplementation (20). The supplementation of dogs' diets with *A. nodosum* also resulted in upregulation of the selenoamino acid metabolism pathway, which was probably associated with the observed significant change in selenomethionine Se-oxide level. The numbers of metabolites belonging to androgen and estrogen biosynthesis and metabolism pathways were also of interest, as was the disappearance of prostaglandin formation from the arachidonate pathway after 30 days of *A. nodosum* supplementation. The lack of dehydroepiandrosterone and testosterone was of particular interest. In humans increased dehydroepiandrosterone is correlated with periodontitis level (21), so in the current study the lack of dehydroepiandrosterone in the saliva of dogs administered *A. nodosum* could be a reflection of improved oral health status; but this is highly speculative.

There are almost no data about saliva composition in healthy or ill dogs. In a recent study, dog saliva metabolomes were compared to human saliva metabolomes (19). The unique canine metabolites identified in that study were mostly lipids and lipid-like molecules (i.e., carnitines). In the current study the presence of L-palmitoylcarnitine was detected in the A. nodosum-treated group. Turunen et al. (19) detected sphinganine in dog saliva but not human saliva, and in the present study, sphinganine-1-phosphate was detected in the *A. nodosum*-treated group. These are two examples of possible concordance between the results of the two studies, but what must be noted—and was also pointed out by (19)—is that thousands of molecules detected results in hundreds of identifications. Thus, specific conclusions are not justified based on currently identified molecules.

The Authors of the current study are also aware of its limitations, especially in the field of metabolites identification. The identification of metabolites was carried out on the basis of the database indications above 80% probability of confirming a given structure. Molecules were identified only based on their molecular weight. Our metabolomic analysis used the MetaboAnalyst software to assign metabolites important in our analysis to well-described metabolic pathways. This analysis identified potential metabolites in a given metabolic pathway. Repetitions of m/z may be due to the involvement of metabolites with the same m/z in two different metabolic pathways. However, our studies were rather focused on showing the differences between the T0 vs. T30 groups and certainly the results of these analyses would require further confirmation of the validity of the data indicated. That is why in the Supplementary Table 1 we included all the raw data from the experiment which could help the readers to validate the results obtained.

In conclusion, the positive clinical effect of 30 days of *A. nodosum* supplementation on oral health status in dogs combined with the absence of many metabolites in the saliva of dogs on day 30 of supplementation suggest that brown algae inhibit or turn off some pathways that could lead to plaque or calculus development. Notably however, it cannot be determined whether these absences are a direct consequence of *A. nodosum* action or the influence of the extract on associated processes in the oral cavity. Metabolomic analyses identified clear changes after 30 days of supplementation, and the direction of these changes was completely different than in dogs that received a placebo treatment during the same period. The exact mechanism of *A. nodosum* is still unclear and warrants further study, but such studies must include analysis of dog saliva composition.

## Data Availability Statement

The original contributions presented in the study are included in the article/Supplementary Material, further inquiries can be directed to the corresponding authors.

## Ethics Statement

All procedures performed during the study were conducted in accordance with standard veterinary care protocols and relevant Polish regulations (Art.1 ust.2 pkt1 Dz. U.2015 poz. 266), so the study did not require ethics committee approval. All dog owners provided written informed consent for the participation of their dogs in the study. Written informed consent was obtained from the owners for the participation of their animals in this study.

## Author Contributions

JG, MJ, and US designed the scientific project. JW performed metabolomic tests and contributed in analyze of the results. JG performed research in animals. MJ made statistics. US helped editing. All authors contributed to the article and approved the submitted version.

## Conflict of Interest

US is employed as a consultant and CEO at the company UKS Life Science AB. The remaining authors declare that the research was conducted in the absence of any commercial or financial relationship that could be construed as a potential conflict of interest.
